# Malingering Detected by Faradisation and the Use of Nitrous Oxide Gas

**Published:** 1872-10

**Authors:** Julius Althaus

**Affiliations:** Physician to the Infirmary for Epilepsy and Paralysis.


### ARTICLE VI.

### *Malingering Detected by Faradisation and the Use of Nitrous Oxide Gas.*

BY JULIUS ALTHAUS, M. D., M. R. C. P.

Physician to the Infirmary for Epilepsy and Paralysis.

In November last I was consulted by the Secretary of a working men's benefit society with regard to the case of one of the society's members, who professed to have lost the use of his left arm, in consequence of an accident which he had had three years previously. According to the society's rules, the sum of £100 is paid to members who are permanently incapacitated for work by disease or accident. The patient had had a fall from a considerable height, and asserted that ever since he had been unable to use his arm. He had been admitted into a provincial hospital, where he remained for three months, and where (to use his own words) "the surgeon tried as hard as he could to cure him," but failed. In course of time the patient, who was known not to have done any work since the occurrence of the accident, applied to his society for the hundred pounds owing to him; and I was then requested to give an opinion whether the patient was permanently or only temporarily disabled.

The claimant was a tall, powerful man of determined countenance, and evidently considerable force of will. He professed to be unable to undress himself, and had, therefore, to be assisted when the helpless limb was bared for examination. I found that the temperature and the bulk of the left arm were in all its parts equal to those of the right. The limb was held in full extension and drawn to the body; while the fingers were somewhat tightly fixed. On endeavoring to flex the forearm and to supinate the hand, considerable resistance was encountered; and when additional force was used for effecting this purpose, the patient called out with pain, and said he could not bear the manipulation.

Seeing this condition of the limb, only three pathological conditions could be suspected, viz., paralysis with contraction, ankylosis, or dislocation—provided always that the patient was sincere. In peripheral paralysis owing to injury of the motor nerves of the part, which is the only form of paralysis that could be thought of in this case, there is rarely any very great amount of contraction, since the paralysed limbs are mostly found flabby; and if the case be of long standing, the muscles are wasted, and the temperature is considerably diminished. But as these clinical signs, although of value, are yet not invariably present, I employed a test which gives absolutely decisive results in such cases, and enables us at a glance to decide the presence or absence of peripheral paralysis—viz., faradisation. It has been shown by the concurrent testimony of all recent observers who have investigated this subject, that in peripheral paralysis caused by injury to the motor nerves, the muscles animated by those nerves completely lose their *faradic* excitability, while their *galvanic* excitability may be preserved, or, under certain circumstances, even increased. If, therefore, in the present case, the deltoid, triceps, biceps, and the other muscles of the useless limb, could be made to respond by contraction to the faradic current, it would be rendered evident that there was no paralysis owing to injury of the branchial plexus or any of its branches.

On using faradisation, I found that all the muscles of the arm and hand responded readily to the current by contraction of their fibres: yet, curiously enough, the arm of the patient did not execute those movements which are generally produced by such an application. Something evidently resisted the displacement of the bones; and when I looked at the powerful determination visibly expressed in every feature of the patient's face, his hard stare, his contracted brow and lips, I could not help feeling suspicious that this something might be the patient's own volition. The influence of faradisation being irresistible if a sufficiently strong current be used, I increased the power with which I acted, in order to overcome any possible resistance on the part of the patient; but the latter called so lustily, saying he could not bear the pain, that I was obliged to desist. Enough, however, had been ascertained for enabling me to eliminate one of the three pathological conditions which could give rise to the complaint of the patient.

I now informed the secretary that, although I had satisfied myself that there was no paralysis, yet it was impossible for me to give a certificate concerning the exact nature of the affection from which the patient suffered, unless he were previously placed under the influence of an anæsthetic. All parties having consented that this should be done, I procured the assistance of Mr. Clover, who on the following day administered nitrous oxide gas to the patient. The latter was rapidly rendered insensible; and I could now freely move the arm in all directions, there being neither dislocation nor ankylosis. As soon as this was ascertained, the influence of the anæsthetic was withdrawn; and the patient, who recovered himself in a few moments, was informed that his case was not nearly so bad as he had imagined, and that he would certainly recover the use of the arm under proper treatment. I gave a certificate to the effect that the patient suffered from a painful affection of the shoulder-joint which would yield rapidly to subcutaneous injections of morphia or a judicious use of galvanism; and that there was neither paralysis, nor dislocation, nor ankylosis, seriously to interfere with the use of the extremity. The claim was therefore not allowed.—*British Med. Journal.*

